# Protective Effect of Saccharides on Freeze-Dried Liposomes Encapsulating Drugs

**DOI:** 10.3389/fbioe.2019.00424

**Published:** 2019-12-17

**Authors:** Diana Guimarães, Jennifer Noro, Carla Silva, Artur Cavaco-Paulo, Eugénia Nogueira

**Affiliations:** Centre of Biological Engineering, University of Minho, Braga, Portugal

**Keywords:** liposomes, freeze-drying, cryo/lyoprotectants, saccharides, drug delivery

## Abstract

The production of freeze-dried liposomes encapsulating drugs is considered a key challenge since the drugs are prone to leakage. The aim of this work was to study the effect of different saccharides on preserving the stability and drug retention capacity of a previously developed liposomal formulation, when subjected to a freeze-drying process. The protective role of trehalose, lactose, glucose, mannitol and sucrose, known for their cryo/lyoprotective effect, was tested by addition of different concentrations to liposomes. Sucrose, in a concentration dependent manner (8:1 sugar:lipids mass ratio) proved to be a suitable cryo/lyoprotectant of these liposomes. Effectively, this saccharide prevents the fusion or/and aggregation of the liposomal formulation, protecting the integrity of the freeze-dried empty liposomes. The liposomal formulation containing sucrose was studied in terms of morphology, concentration, and anticancer drugs retention ability. The study involved two drugs encapsulated in the aqueous core, methotrexate (MTX) and doxorubicin (DOX), and one drug located in the lipid bilayer, tamoxifen (TAM). After the freeze-drying process, liposomes with sucrose encapsulating drugs revealed high physical stability, maintaining their narrow and monodisperse character, however high leakage of the drugs encapsulated in the aqueous core was observed. Otherwise, no significant drug leakage was detected on liposomes containing the TAM, which maintained its biological activity after the freeze-drying process. These findings reveal that sucrose is a good candidate for the cryo/lyoprotection of liposomes with drugs located in the lipid bilayer.

## Introduction

Liposomes have received significant attention as drug delivery systems since they are composed of natural substances, making them non-toxic and biodegradable (Nogueira et al., 2015a). Drugs encapsulated within liposomes are protected from early inactivation, immediate dilution or degradation, suggesting these devices as good carriers to targeting sites (Spuch and Navarro, 2011). Depending on their solubility, drugs can be encapsulated in the inner aqueous compartment (hydrophilic drugs), intercalated in the membrane bilayer structure or associated to the membrane surface (hydrophobic drugs) (Schwendener and Schott, 2017). In addition, amphipathic acids or bases can be loaded in the inner aqueous core of liposomes (Haran et al., 1993; Clerc and Barenholz, 1995).

The potential of liposomes for specific therapeutic applications continues to be a challenge due to their inherent physical and chemical instability for long-term storage. Some of the problems include hydrolysis or oxidation of phospholipids, liposome aggregation or/and fusion, and increased bilayer permeability resulting in drug leakage (Payton et al., 2014; Porfire et al., 2017; Olusanya et al., 2018). A usual approach to overcome these problems is the production of a dry liposomal product. Freeze-drying (i.e., lyophilization) is the method commonly used to improve the long-term stability of dry powder liposomes with low content of residual water (Alexopoulou et al., 2006; Kannan et al., 2014). However, freeze-drying itself may result in formulation physical changes, related with the increase of the size of liposomes, resulting from the fusion of the phospholipid membranes that can occur during the freezing, drying, or rehydration. The loss of the encapsulated drug is also a very common drawback related with this process (Van Winden, 2003). Therefore, a meticulous optimization and selection of the components of the formulation is crucial to achieve a long-term stability of liposome based drugs (Kannan et al., 2014).

The inclusion of cryo and lyoprotectants in the liposomal formulation has been undertaken to improve the functional properties and stability of the products after freezing and drying, respectively (Costantino et al., 2004). Since they show the ability to act as the integrity membrane protectants, carbohydrates, more specific the saccharides, are the preferable cryo/lyoprotectants used during dehydration/rehydration of liposomes (Hua et al., 2003; Sylvester et al., 2018). To achieve a highly stable liposomal formulation some aspects must be optimized, namely the type and concentration of saccharide. Some theories have been proposed to explain the stabilization mechanisms beyond the use of cryo/lyoprotectants during freeze-drying (Ingvarsson et al., 2011; Franzé et al., 2018), however deeper studies must be undertaken to understand their protective effect on the freeze-dried liposomes.

We have previously described a liposomal formulation that prove to be an efficient system for the encapsulation and delivery of both hydrophobic and hydrophilic drugs (Nogueira et al., 2015c). The results support their use as therapeutic delivery systems as demonstrated by the biological effect of several drugs *in vitro* as well as *in vivo* (Nogueira et al., 2015b,c). The aim of this work was to optimize this liposomal formulation to preserve its initial characteristics upon hydration of freeze-dried powder. For this, we introduced in the liposome suspension different concentrations of saccharides, namely trehalose, lactose, glucose, mannitol and sucrose, and freeze-dried the final formulation. We foresee that these new formulations, similarly to the raw formulation, will give rise to stable and homogeneous suspensions with a minimum of drug leakage. The physicochemical properties (like size, morphology, and concentration) of the optimized liposomal formulations were investigated. Furthermore, the influence of the overall freeze-drying process on the leakage of three different anticancer drugs was explored and the biological activity of the most promising liposomal formulation was determined.

## Materials and Methods

### Materials

1,2-dioleoyl-sn-glycero-3-phosphoethanolamine (DOPE) and 1,2- distearoyl-*sn*-glycero- 3-phosphoethanolamine-N-[methoxy (polyethylene glycol)-2000] (DSPE–MPEG) were obtained from Lipoid GmbH (Germany). Deuterium oxide and deuterated chloroform were purchased from Cortecnet. Cholesterol (CH), methotrexate (MTX), doxorubicin hydrochloride salt (DOX), tamoxifen (TAM), pyridine as well as the saccharides (trehalose, lactose, glucose, mannitol and sucrose) were received from Sigma-Aldrich (USA). All culture media and supplements were also purchased from Sigma-Aldrich (USA).

### Liposome Preparation

The production of liposomes encapsulating MTX and TAM was performed by passive loading of drugs. Liposomes composed of DOPE/CH/DSPE-mPEG (54:36:10, molar ratio) (Nogueira et al., 2015c) were produced by ethanol injection method (Jaafar-Maalej et al., 2010). Briefly, an amount of DOPE, CH and DSPE-mPEG was dissolved in ethanol. The organic phase was injected under vigorous magnetic stirring to aqueous phase, phosphate-buffered saline (PBS) buffer (pH 7.4), at 70°C. When indicated, sucrose was dissolved in PBS buffer at 8 g/g of dry lipids, to be present in both sides of liposomes. The vesicles were then extruded (extruder supplied by Lipex Biomembranes Inc., Vancouver, Canada) several times through polycarbonate filters of 200 nm and after 100 nm pore size (Nucleopore) to form unilamellar vesicles. Encapsulation of drugs was done by their mixture during the liposomes preparation, MTX (7 mg/mL), as hydrophilic drug was included in aqueous phase (PBS buffer) and TAM (1 mg/mL) as hydrophobic drug was added in organic phase (ethanol). MTX disodium salt was prepared adding two NaOH molar equivalent to a PBS buffer containing commercial MTX. After completely solubilization of MTX, the pH was adjusted to pH 7.4.

The production of liposomes encapsulating DOX was obtained by active loading. Empty liposomes were prepared as described above, by ethanol injection method, using 120 mM ammonium sulfate buffer (pH 8.5) as aqueous phase, instead of PBS buffer. After extrusion, the buffer was exchanged in a Sephadex PD-10 desalting column (GE Healthcare, UK), equilibrated with 25 mM Tris Base sucrose (10%, w/v, pH 9.0). Remote loading of DOX (2.5 mg/mL) was carried out through ammonium sulfate gradient approach, upon incubation with liposomes for 1.5 h at 60°C (Haran et al., 1993).

### Determination of Drug Encapsulation and Leakage

The non-encapsulated drugs were removed from the liposomes after passage through a gel filtration chromatography column (GE Healthcare, UK), with 5 kDa cut-off (PD-10 Desalting columns containing 8.3 mL of Sephadex™ G-25 Medium) and eluted with PBS buffer for all liposome. The concentration of MTX and TAM encapsulated was measured by proton nuclear magnetic resonance (^1^H NMR) using a Bruker Avance III Instrument, operating at 400 MHz (Guimarães et al., 2019). Powder liposomes containing drug were dissolved in deuterium oxide (for MTX) or deuterated chloroform (for TAM) to determine the amount of drug in the liposomal formulation. Pyridine was used as internal standard. Quantification of DOX was evaluated by UV–vis spectrophotometry measuring the absorbance at 490 nm. UV–vis spectra of liposomes encapsulated DOX were recorded on spectrophotometer BioTek Synergy™ HT using a plastic microplate. The final DOX concentration was determined based on the respective calibration curve.

The drug leakage was determined as follows:

$$\begin{array}{l} \%\text{ Leakage}=\frac{\left[\text{Drug}\right]\text{ encapsulated after freeze drying}}{\;\left[\text{Drug}\right]\text{ encapsulated before freeze drying}}\;\times \;100 \end{array}$$

### Determination of Size Distribution

The physicochemical characterization of liposomes was evaluated in terms of size and polydispersity index (PDI) using the dynamic light scattering technique (DLS) (at least three replicates for each formulation). The analysis was determined at pH 7.4 (PBS buffer) and at 25.0°C, using a Malvern Zetasizer Nano ZS (Malvern Instruments) by photon correlation spectroscopy (PCS). The viscosity and refractive index values used were 0.8616 cP and 1.332, respectively.

### Freeze-Drying and Liposomes Hydration

The saccharides used in this work (trehalose, lactose, glucose, mannitol, and sucrose) were dissolved in PBS buffer at different concentrations of 2, 4, 6, and 8 g/g of dry lipids. Liposomal suspensions were diluted in an equal volume of each saccharide buffered solution in 50 mL tubes, at 10% of fill volume. As control, liposomal suspension was diluted in equal volume of PBS buffer. A very low freezing temperature seems to avoid damage of nanoparticles and improve the lyoprotective effect, as demonstrated in previous studies using nanoparticles with saccharides (De Jaeghere et al., 2000; Moretton et al., 2012). Considering these assumptions, all the liposomal suspensions were stored for 6 h at −80°C in a deep freezer and then freeze-dried. When indicated, liposomes were stored in a Corning® CoolCell™, in order to achieve a slow rate of freezing, ~ −1°C/min. The freeze-drying process was performed involved only the primary drying using the Labconco FreeZone 2.5 freeze dryer (Labconco, Kansas City, USA), for 24 h at −50°C in a chamber with 6 Pa (conditions set by the equipment). The reconstitution of freeze-dried liposomes to their original volume was made at room temperature with PBS using a vortex mixer. The samples were equilibrated for 1 h and subjected to further tests.

### Nanoparticle Tracking Analysis

Nanoparticle tracking analysis (NTA) measurements were performed using a NanoSight NS500 instrument (Salisbury, UK) equipped with a charge coupled device (CCD) camera that allows the visualization and tracking Brownian motion of laser-illuminated particles in suspension. The measurements were made at room temperature and each video sequence was captured over 60 s with manual shutter and gain adjustments. The samples were diluted with water and then injected into the system (at least three replicates for each formulation).

### Thermogravimetric Analysis

Thermogravimetric analysis (TGA) was performed in a TGA 4000 (Perkin Elmer, Waltham, MA, US) using an alumina crucible with sample weights of approximately 25 mg. The temperature calibration was established by Curie temperatures of reference materials: alumel, nickel, and perkalloy at the analysis scanning rate. The measurements were performed from 25 to 800°C at 10°C/min under a nitrogen atmosphere (flow rate: 20 mL/min). The weight loss percentage and its derivative were represented as function of temperature. Derivative weight percentages were used to measure the weight loss as function of temperature (establish the start and end of each degradation step) and compare the peak temperatures. The weight loss was used to calculate the total amount of residual water.

### Microscopy Imaging Analysis

The morphology of liposomes was evaluated by scanning transmission electron microscopy *(*STEM). The initial liposomes suspensions and after freeze-drying with sucrose (8:1 mass ratio) were dropped in Copper grids with carbon film 400 meshes, 3 mm diameter. The shape and morphology of nanoparticles were observed using a NOVA Nano SEM 200 FEI system.

### Cell Culture Conditions

The human breast adenocarcinoma cell line MCF-7 (ATCC® HTB-22™) was obtained from the American Type Culture Collection. Cells were maintained in Roswell Park Memorial Institute (RPMI) 1640 medium supplemented with 2.0 g/L sodium bicarbonate, 10 mM HEPES, 10% (v/v) of fetal bovine serum (FBS), 1% (v/v) penicillin/streptomycin solution and 0.01 mg/mL insulin. Cells were grown in T75 flasks (SPL Life sciences, Korea) and maintained at 37°C in a humidified atmosphere of 5% CO_2_. MCF-7 cells were routinely sub-cultured two times a week.

### Metabolic Activity Assay

Metabolic activity was studied using a 3-(4,5-dimethylthiazol-2-yl)-5-(3-carboxymethoxyphenyl)-2-(4-sulfophenyl)-2H- tetrazolium (MTS) assay. A ready-for-use CellTiter 96® Aqueous One solution of MTS (Promega, Madison, USA) was used according to the protocol suggested by the manufacturer. Cells were seeded at a density of 1.5 × 10^4^ cells per 100 μL/well on 96-well TCPS plates (TPP, Switzerland) in the day before the experiment to promote cell adhesion. The cells were incubated for 48 h with then exposed to different liposome and drug concentrations (three replicates for each condition). After this time, the culture medium was refreshed, and 20 μL of CellTiter 96® Aqueous One solution was added to each well. After 4 h of incubation at 37°C, the absorbance at 490 nm was measured using a microplate reader (Synergy Mx Multi-Mode Reader, BioTek, USA). Metabolic activity was expressed as a percentage relative to the negative control (untreated control cells).

### Statistical Analysis

Statistical analyses were performed with GraphPad Prism software (version 5.0). Differences were tested for statistical significance by a one-way Analysis of variance (ANOVA).

## Results and Discussion

### Influence of Saccharides on Liposomes' Size Distribution

**Figure 1:**
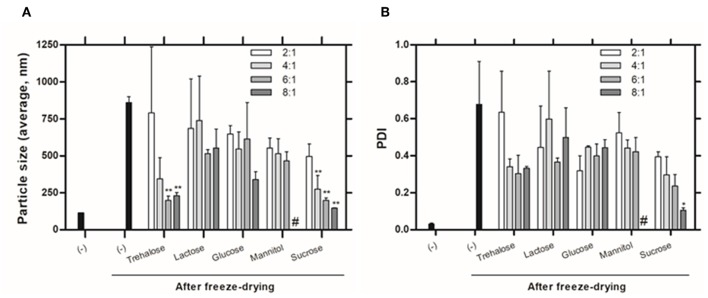
Physicochemical characteristics of liposomes evaluated by DLS: **(A)** Size (Z-average) and **(B)** PDI of liposomes with (w:w, sugar:lipids) and without (-) saccharides, before and after freeze-drying. # Values not determined due to non-homogenous dispersion obtained. Values represent the mean + SD of 2 independent experiments. Significant differences between liposomes without and with saccharides were detected as shown by an *P < 0.05 and **P < 0.005.

The preservation of the physical integrity of liposomes during freeze-drying process it is the primordial importance and can be achieved by the inclusion of saccharides at the final formulations. Since the stabilization effect promoted by cryo/lyoprotectants is concentration-dependent (Ball et al., 2017), we compared the protective effect of five saccharides at different concentrations on the final formulation. The fusion or aggregation of liposomes during the freeze-drying process was monitored by measuring the size distribution of liposomes after freeze-drying-rehydration cycle and compared with the results of freshly prepared liposomes. The values of size and PDI of liposomes before and after freeze-drying process are shown in Figures 1A,B, respectively.

The size and PDI of liposomes without saccharides in their composition increase significantly after the freeze-drying process (from 113.8 ± 0.99 nm to 859.0 ± 56.14 nm and from 0.03 ± 0 to 0.57 ± 0.49). One can also observe that the protective effect of the saccharides is concentration-dependent. Inadequate concentration of sugar can lead to incomplete coating of the glassy matrix around nanoparticles promoting aggregation (Date et al., 2010). Smaller particle sizes were obtained by using trehalose and sucrose at higher concentrations, however, only sucrose at 8:1 (sugar:lipids) mass ratio allows to achieve a homogeneous suspension, with PDI around 0.1.

Higher sucrose molar ratios were also tested to decrease the size and polydispersity and meet the initial size of the liposomal formulation without cryo/lyoprotectants (Figure S1). However, we found that higher concentrations of sucrose lead to similar liposomes' size and polydispersity as obtained when using the 8:1 mass ratio.

A few theories have been proposed to explain the mechanism beyond the stabilizing action of cryo/lyoprotectants during the freeze-drying process (Mensink et al., 2017; Franzé et al., 2018). There is no generally accepted theory, being that these compounds exert their action via one or more of the following mechanisms. The water replacement theory attributes the stabilization effect of protectors to their ability to replace the bound water around the bilayers through specific interactions with the polar region of the lipid head group at low hydrations. In the vitrification theory, a highly viscous matrix is formed around the liposome which reduces the mobility during the freeze-drying process (Sun et al., 1996; Chen et al., 2010). Kosmotropic effects, the less common theory, establishes that cryoprotectants interact with water and disrupt their normal structure. The damage during freeze-drying is prevented due to the reduction of water content at membrane interface (Ingvarsson et al., 2011). Despite of the theories, deeper studies must be undertaken to understand their protective effect on the freeze-dried liposomes. However, saccharides such as sucrose or trehalose are described to be very effective lyoprotectants, as they show a very high viscosity, a low molecular mobility after drying and form an amorphous, glassy matrix (Slade and Levine, 1995), which corroborates our findings.

### Residual Water Content

The residual water content of formulations can be one of the most important factors affecting the stability of freeze-dried products. It has been demonstrated that high levels of residual water content lead to a unexpected dissolution of the freeze-dried samples immediately after freeze-drying, or to a poor long-term storage stability of nanoparticles (Fonte et al., 2016). After freeze-drying process, the liposomal formulation must have a water content <2% (Sylvester et al., 2018). The total amount of residual water in liposomes after freeze-drying was herein determined by TGA. All freeze-dried liposomes contained <1.5% of residual water (Figure S2). This very low water content proves the efficiency of primary drying, avoiding an additional secondary drying step.

### Influence of the Freeze-Drying Process on the Liposomes Concentration

Fusion or aggregation of liposomes during freeze-drying process can be monitored by determination of the liposomes concentration. To determine if the freeze-dried liposomes maintain the same concentration as of the initial liposomes, the NTA was performed. The ability of NTA to simultaneously measure size and particle scattering intensity, makes possible the direct estimation of the particles concentration. Furthermore, its ability to determine the size distribution of particles until 2 μm in diameter (according to the manufacturer, Malvern), allow us to evaluate liposomes in a micrometer range. A lower concentration of particles was observed for freeze-dried liposomes without cryo/lyoprotectants (Table 1). These results are in good agreement with the results obtained by DLS, which revealed higher particle sizes, probably due to fusion or aggregation phenomena. After freeze-drying the concentration of liposomes containing sucrose remained similar to the initial formulation concentration, highlighting the protective role of the cryo/lyoprotectant.

**Table 1:** Influence of freeze-drying on liposomes concentration, determined by NTA.

**Sample**	**Freeze drying**	**Concentration** **(E^**12**^ particles/mL)**
Liposomes	-	20.1 ± 3.1
	+	10.3 ± 2.1
Liposomes + sucrose	-	23.3 ± 7.8
	+	21.3 ± 3.9

*Values represent the mean of ± SD of 3 independent experiments*.

### Morphology of Liposomes After Freeze-Drying

**Figure 2:**
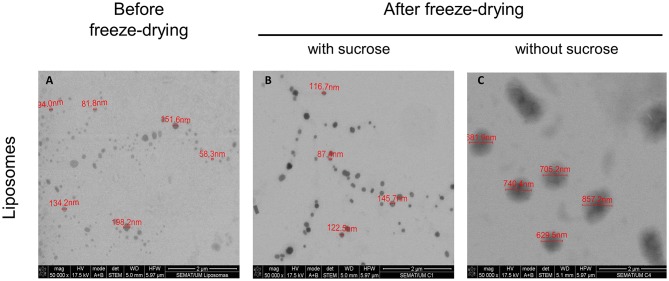
Morphology profile of liposomes. Representative STEM images of liposomal formulation **(A)** before freeze-drying, **(B)** with sucrose (8:1 mass ratio), after freeze-drying and **(C)** without sucrose, after freeze-drying. The scale bar in the figures represents 2 μm.

The morphology of particles is of outmost importance to identify possible fusion and/or aggregation phenomena. Liposome suspensions, before and after freeze-drying, were observed by STEM. The profile of the initial liposomal formulation (Figure 2A) and of the freeze-dried liposomes containing sucrose (8:1 sugar:lipids, mass ratio) as cryo/lyoprotectant (Figure 2B), revealed few fused or aggregated particles, being the liposomes in the form of individual vesicles. Furthermore, the liposomal formulation presents a homogeneous population with spheroid and regular shape. The images show that freeze-dried liposomes containing sucrose were stable revealing no significant increase of the particle size and maintaining their spherical shape. This morphology can offer potential for controlled release and protection of incorporated drugs, as they provide minimum contact with the aqueous environment favoring a longest diffusion pathway. The result are in agreement with the DLS data showing a very similar particle size distribution. Significant changes in the lipid aggregate structure and size was noted upon rehydration of freeze-dried liposomes without the protective effect of sucrose (Figure 2C). Liposomes exhibit fused or aggregated vesicles, presenting some cylinder-like shape particles. In addition, a distinct population of significantly larger liposomes was detected in the samples. These observations are also supported by DLS data.

### Influence of the Freeze-Drying on Drug Leakage From Liposomes

Freeze-drying is an approach to dry liposomal formulations well-established by the pharmaceutical industry to improve the long-term storage stability of drugs. However, the stresses imposed during the freeze-drying process might lead to the leakage of the encapsulated drugs (Ingvarsson et al., 2011). The drug leakage depends on the liposome composition and on the nature of drug (Pauli et al., 2019). Thus, it is imperative to study the freeze-drying process on drug leakages, and evaluate their influence on the final liposomal formulations behavior.

Leakage of three anticancer drugs was evaluated only in liposomes with sucrose (8:1 sugar:lipids, mass ratio), since liposomes without cryo/lyoprotectant have a high values of size and PDI. MTX, a hydrophilic drug in a disodium salt form, was encapsulated into the aqueous core of liposomes and their behavior after the freeze-drying process was investigated. The results showed that despite the presence of sucrose, the leakage of MTX was very high after freeze-drying process (61.1%, Table 2). From the data obtained (Table S1), higher leakage levels remained even after using a slow rate of freezing (~ −1°C/min) and the distribution of the cryo/lyoprotetor in both sides of liposomes (outer and inner part). We also evaluated the behavior of different drug:lipid molar ratio, however high leakages levels were also observed (data not shown). It is noteworthy that buffer pH changes might influence drug leakage. For this reason, the pH of liposomes encapsulating MTX (in PBS buffer) was measured and did not showed any alteration after freezing and drying processes.

**Table 2:** Characterization of liposomes before freeze-drying and after freeze-drying with addiction of sucrose.

	**Before freeze-drying**				**After freeze-drying**		
**Encapsulated drug**	**Z-average (d.nm)**	**PDI**	**Encapsulation efficiency (%)**	**Drug:lipid (molar ratio)**	**Z-average (d.nm)**	**PDI**	**Leakage (%)**
(-)	125.5 ± 4.3	0.068 ± 0.036	-	-	154.3 ± 1.9	0.135 ± 0.009	-
MTX	126.5 ± 3.5	0.078 ± 0.008	2.6 ± 0.1	~1:11	166.5 ± 4.1	0.165 ± 0.053	61.1 ± 4.0
DOX	134.1 ± 0.5	0.111 ± 0.018	65.3 ± 1.4	~1:11	169.1 ± 3.4	0.200 ± 0.012	24.5 ± 0.3
TAM	107.8 ± 1.4	0.036 ± 0.003	93.9 ± 6.1	~1:11	148.5 ± 4.4	0.130 ± 0.020	4.0 ± 3.0

*Values represent the mean ± SD of 3 independent experiments*.

The influence of the preparation method of liposomes was also evaluated using the remote loading. However, this approach can only be applied to weak amphipathic acids or bases, which MTX molecule does not belong to Alekseeva et al. (2017). Liposomes encapsulating DOX, prepared by remote loading, was then used in this study, however, high leakage level is also observed (24.5%, Table 2). These results indicate that the leakage is not directly governed by the loading method, but possibly by the location of drug in liposomes.

This behavior might be justified by the presence of unsaturated phospholipid DOPE (Monteiro et al., 2014) in the liposomal composition. However, its presence in the optimal liposomal formulation is crucial to obtain pH-sensitive devices which, in therapeutic applications, facilitate the release of drugs into the cell cytoplasm. The drug leakage of drugs encapsulated in the aqueous core may be also enhanced by eventual bilayer defects (Nogueira et al., 2016). These effects can cause a change in the pharmacokinetic profile of the encapsulated drug and lead to reduce the reproducibility of the therapeutic effect.

To study the behavior of a drug located in the lipid bilayer, TAM was also encapsulated into liposomes. The results obtained indicate that freeze-dried liposomes containing sucrose with TAM encapsulated have lower drug leakage (4.0%, Table 2). As performed for liposome encapsulating MTX, the pH of formulations containing TAM was also evaluated and no alterations were observed after freezing and drying processes. The pattern of size distribution is similar to the empty liposomes, showing small increase of size and PDI. These liposomes are stable for at least 3 months after rehydration (data not shown).

### Influence of Freeze-Drying on the Biological Activity of Liposomes Encapsulating Tamoxifen

**Figure 3:**
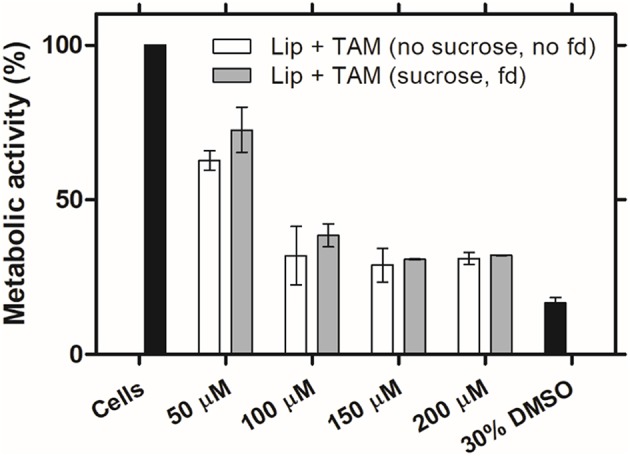
Biologic activity of liposomes encapsulating TAM after freeze-drying process. MCF-7 cell line metabolic activity after 48 h of incubation with the initial liposomal formulations (without sucrose) and after freeze-drying (fd) with sucrose (8:1 sugar:lipids, mass ratio), at different concentrations of TAM. Values represent the mean ± SD of 2 independent experiments.

It is of prime importance that a therapeutic drug preserve its functionality and biological activity, even after the freeze-drying process. In this way, liposomes encapsulating TAM are expected to present the same biological activity profile after the freeze-drying process. TAM, as a selective estrogen receptor (ER) modulator, is indicated for the treatment of breast cancer ER-positive (Nazarali and Narod, 2014). As a hydrophobic drug, TAM may induce oxidative stress due to the accumulation in phospholipid bilayers of membranes resulting in cell death at highest concentrations (Gundimeda et al., 1996). The effect of freeze-drying process on TAM biological activity was evaluated *in vitro* using MCF-7 cell line, an ER-positive breast cancer cell line (Pawlik et al., 2016). It was assessed the metabolic activity of cells after the incubation with different concentrations of TAM encapsulated into liposomes, before and after the freeze-drying process. Based on the collected data at 48 h, liposomes encapsulating TAM after freeze-drying process did not exhibit significant loss of biological activity compared to liposomes before freeze-drying (Figure 3). These results confirm that the freeze-drying process does not affect the biological activity of the drug.

## Conclusion

We have successfully optimized a liposomal formulation by incorporation of a sugar, sucrose, which preserved its integrity after the freeze-drying process. From all the saccharides tested, only sucrose at 8:1 mass ratio presented ability to protect the dry liposomal formulation. The size distribution, morphology analysis and concentration of the final formulations indicate that liposomes are not subject to fusion or/and aggregation. However, leakage of drugs encapsulated in the aqueous core, MTX and DOX, after freeze-drying process is observed, even though the different preparation method used (passive and active loading, respectively). Otherwise, liposomes with a drug located in the lipid bilayer, TAM, demonstrated negligible leakage preserving their biological activity after the freeze-drying process. In this way, leakage seems to be dependent of location of the drug in liposomes.

Taken together, the results indicated that sucrose protects the physical and biological integrity of the liposomal formulations encapsulating TAM when exposed to freeze-drying process being a promising approach for storage.

## Data Availability Statement

All datasets generated for this study are included in the article/Supplementary Material.

## Author Contributions

DG and EN designed the studies, analyzed the data, and wrote the paper. DG performed the experiments. JN contributed to the analyses of ^1^H NMR. CS supported the NTA experiments and enhanced the paper. EN and AC-P assisted in the preparation of the manuscript. All authors read and commented on the manuscript.

### Conflict of Interest

The authors declare that the research was conducted in the absence of any commercial or financial relationships that could be construed as a potential conflict of interest.

## References

[B1] Alekseeva A. A. Moiseeva E. V. Onishchenko N. R. Boldyrev I. A. Singin A. S. Budko A. P.. (2017). Liposomal formulation of a methotrexate lipophilic prodrug: assessment in tumor cells and mouse T-cell leukemic lymphoma. Int. J. Nanomed. 12, 3735–3749. 10.2147/IJN.S133034 28553111 PMC5439940

[B2] Alexopoulou E. Georgopoulos A. Kagkadis K. A Demetzos C. (2006). Preparation and characterization of lyophilized liposomes with incorporated quercetin. J. Liposome Res. 16, 17–25. 10.1080/08982100500528594 16556547

[B3] Ball R. L. Bajaj P. Whitehead K. A. (2017). Achieving long-term stability of lipid nanoparticles: examining the effect of pH, temperature, and lyophilization. Int. J. Nanomed. 12, 305–315. 10.2147/IJN.S123062 28115848 PMC5221800

[B4] Chen C. Han D. Cai C. Tang X. (2010). An overview of liposome lyophilization and its future potential. J. Control Release 142, 299–311. 10.1016/j.jconrel.2009.10.024 19874861

[B5] Clerc S. Barenholz Y. (1995). Loading of amphipathic weak acids into liposomes in response to transmembrane calcium acetate gradients. Biochim. Biophys. Acta 1240, 257–265. 10.1016/0005-2736(95)00214-6 8541297

[B6] Costantino Henry R. Michael J. Pikal P. D. (2004). Lyophilization of Biopharmaceuticals. Arlington, VA: AAPS Press.

[B7] Date P. V. Samad A. Devarajan P. V. (2010). Freeze thaw: a simple approach for prediction of optimal cryoprotectant for freeze drying. AAPS PharmSciTech 11, 304–313. 10.1208/s12249-010-9382-3 20182826 PMC2850490

[B8] De Jaeghere F. Allémann E. Feijen J. Kissel T. Doelker E. Gurny R. (2000). Freeze-drying and lyopreservation of diblock and triblock poly(lactic acid)-poly(ethylene oxide) (PLA-PEO) copolymer nanoparticles. Pharm. Dev. Technol. 5, 473–483. 10.1081/PDT-100102031 11109247

[B9] Fonte P. Reis S. Sarmento B. (2016). Facts and evidences on the lyophilization of polymeric nanoparticles for drug delivery. J. Control Release 225, 75–86. 10.1016/j.jconrel.2016.01.034 26805517

[B10] Franzé S. Selmin F. Samaritani E. Minghetti P. Cilurzo F. (2018). Lyophilization of liposomal formulations: still necessary, still challenging. Pharmaceutics 10:139. 10.3390/pharmaceutics10030139 30154315 PMC6161153

[B11] Guimarães D. Noro J. Loureiro A. Cavaco-Paulo A. Nogueira E. (2019). Quantification of drugs encapsulated in liposomes by 1 H NMR. Colloids Surfaces B Biointerfaces 179, 414–420. 10.1016/j.colsurfb.2019.03.039 30999120

[B12] Gundimeda U. Chen Z. H. Gopalakrishna R. (1996). Tamoxifen modulates protein kinase C via oxidative stress in estrogen receptor-negative breast cancer cells. J. Biol. Chem. 271, 13504–13514. 10.1074/jbc.271.23.13504 8662863

[B13] Haran G. Cohen R. Bar L. K. Barenholz Y. (1993). Transmembrane ammonium sulfate gradients in liposomes produce efficient and stable entrapment of amphipathic weak bases. Biochim. Biophys. Acta 1151, 201–215. 10.1016/0005-2736(93)90105-9 8373796

[B14] Hua Z.-Z. Li B.-G. Liu Z.-J. Sun D.-W. (2003). Freeze-drying of liposomes with cryoprotectants and its effect on retention rate of encapsulated ftorafur and vitamin A. Dry. Technol. 21, 1491–1505. 10.1081/DRT-120024489

[B15] Ingvarsson P. T. Yang M. Nielsen H. M. Rantanen J. Foged C. (2011). Stabilization of liposomes during drying. Expert Opin. Drug Deliv. 8, 375–388. 10.1517/17425247.2011.553219 21294603

[B16] Jaafar-Maalej C. Diab R. Andrieu V. Elaissari A. Fessi H. (2010). Ethanol injection method for hydrophilic and lipophilic drug-loaded liposome preparation. J. Liposome Res. 20, 228–243. 10.3109/08982100903347923 19899957

[B17] Kannan V. Balabathula P. Thoma L. A. Wood G. C. (2014). Effect of sucrose as a lyoprotectant on the integrity of paclitaxel-loaded liposomes during lyophilization. J. Liposome Res. 0, 1–9. 10.3109/08982104.2014.992023 25534990

[B18] Mensink M. A. Frijlink H. W. van der Voort Maarschalk K. Hinrichs W. L. J. (2017). How sugars protect proteins in the solid state and during drying (review): mechanisms of stabilization in relation to stress conditions. Eur. J. Pharm. Biopharm. 114, 288–295. 10.1016/j.ejpb.2017.01.024 28189621

[B19] Monteiro N. Martins A. Reis R. L. Neves N. M. (2014). Liposomes in tissue engineering and regenerative medicine. J. R. Soc. Interface 11:20140459. 10.1098/rsif.2014.0459 25401172 PMC4223894

[B20] Moretton M. A. Chiappetta D. A. Sosnik A. (2012). Cryoprotection-lyophilization and physical stabilization of rifampicin-loaded flower-like polymeric micelles. J. R. Soc. Interface 9, 487–502. 10.1098/rsif.2011.0414 21865255 PMC3262430

[B21] Nazarali S. A. Narod S. A. (2014). Tamoxifen for women at high risk of breast cancer. Breast Cancer 6, 29–36. 10.2147/BCTT.S43763 24648767 PMC3933348

[B22] Nogueira E. Cruz C. F. Loureiro A. Nogueira P. Freitas J. Moreira A.. (2016). Assessment of liposome disruption to quantify drug delivery *in vitro*. Biochim. Biophys. Acta 1858, 163–167. 10.1016/j.bbamem.2015.11.008 26589183

[B23] Nogueira E. Gomes A. C. Preto A. Cavaco-Paulo A. (2015a). Design of liposomal formulations for cell targeting. Colloids Surf. B Biointerfaces 136, 514–526. 10.1016/j.colsurfb.2015.09.034 26454541

[B24] Nogueira E. Lager F. Le Roux D. Nogueira P. Freitas J. Charvet C.. (2015b). Enhancing methotrexate tolerance with folate tagged liposomes in arthritic mice. J. Biomed. Nanotechnol. 11, 2243–2252. 10.1166/jbn.2015.2170 26510317

[B25] Nogueira E. Mangialavori I. C. Loureiro A. Azoia N. G. Sárria M. P. Nogueira P.. (2015c). Peptide anchor for folate-targeted liposomal delivery. Biomacromolecules 16, 2904–2910. 10.1021/acs.biomac.5b00823 26241560

[B26] Olusanya T. O. B. Ahmad R. R. H. Ibegbu D. M. Smith J. R. Elkordy A. A. (2018). Liposomal drug delivery systems and anticancer drugs. Molecules 23, 1–17. 10.3390/molecules23040907 29662019 PMC6017847

[B27] Pauli G. Tang W. L. Li S. D. (2019). Development and characterization of the solvent-assisted active loading technology (SALT) for liposomal loading of poorly water-soluble compounds. Pharmaceutics 11:465. 10.3390/pharmaceutics11090465 31505795 PMC6781273

[B28] Pawlik A. Słominska-Wojewódzka M. Herman-Antosiewicz A. (2016). Sensitization of estrogen receptor-positive breast cancer cell lines to 4-hydroxytamoxifen by isothiocyanates present in cruciferous plants. Eur. J. Nutr. 55, 1165–1180. 10.1007/s00394-015-0930-1 26014809 PMC4819954

[B29] Payton N. M. Wempe M. F. Xu Y. Anchordoquy T. J. (2014). Long-term storage of lyophilized liposomal formulations. J. Pharm. Sci. 103, 3869–3878. 10.1002/jps.24171 25308534 PMC4441342

[B30] Porfire A. Muntean D. M. Rus L. Sylvester B. Tomutǎ I. (2017). A quality by design approach for the development of lyophilized liposomes with simvastatin. Saudi Pharm. J. 25, 981–992. 10.1016/j.jsps.2017.01.007 29158704 PMC5681309

[B31] Schwendener R. A. Schott H. (2017). Liposome formulations of hydrophobic drugs. Methods Mol. Biol. 1522, 73−82. 10.1007/978-1-4939-6591-5_6 27837531

[B32] Slade L. Levine H. (1995). Glass transitions and water-food structure interactions. Adv. Food Nutr. Res. 38, 103–269. 10.1016/S1043-4526(08)60084-4 15918292

[B33] Spuch C. Navarro C. (2011). Liposomes for targeted delivery of active agents against neurodegenerative diseases (Alzheimer's Disease and Parkinson's Disease). J. Drug Deliv. 2011:469679. 10.1155/2011/469679 22203906 PMC3238398

[B34] Sun W. Q. Leopold A. C. Crowe L. M. Crowe J. H. (1996). Stability of dry liposomes in sugar glasses. Biophys. J. 70, 1769–1776. 10.1016/S0006-3495(96)79740-0 8785336 PMC1225146

[B35] Sylvester B. Porfire A. Van Bockstal P. J. Porav S. Achim M. Beer T.. (2018). Formulation optimization of freeze-dried long-circulating liposomes and in-line monitoring of the freeze-drying process using an NIR spectroscopy tool. J. Pharm. Sci. 107, 139–148. 10.1016/j.xphs.2017.05.024 28551424

[B36] Van Winden E. C. A. (2003). Freeze-drying of liposomes: theory and practice. Methods Enzymol. 367, 99–110. 10.1016/S0076-6879(03)67008-4 14611061

