# Supplementary material for: Protective Effect of Saccharides on Freeze-Dried Liposomes Encapsulating Drugs
Source: Front Bioeng Biotechnol. 2019 Dec 17;7:424. doi: 10.3389/fbioe.2019.00424 (PMC6927910; doi:10.3389/fbioe.2019.00424)
Supplement: Supplementary file 1 [file Data_Sheet_1.docx]

Supplementary Material

Protective effect of saccharides on freeze-dried liposomes encapsulating drugs

Diana Guimarães, Jennifer Noro, Carla Silva, Artur Cavaco-Paulo, Eugénia Nogueira*

Centre of Biological Engineering, University of Minho, Braga, Portugal

* Correspondence to: Eugénia Nogueira

E-mail address: [enogueira@ceb.uminho.pt](mailto:enogueira@ceb.uminho.pt)

**
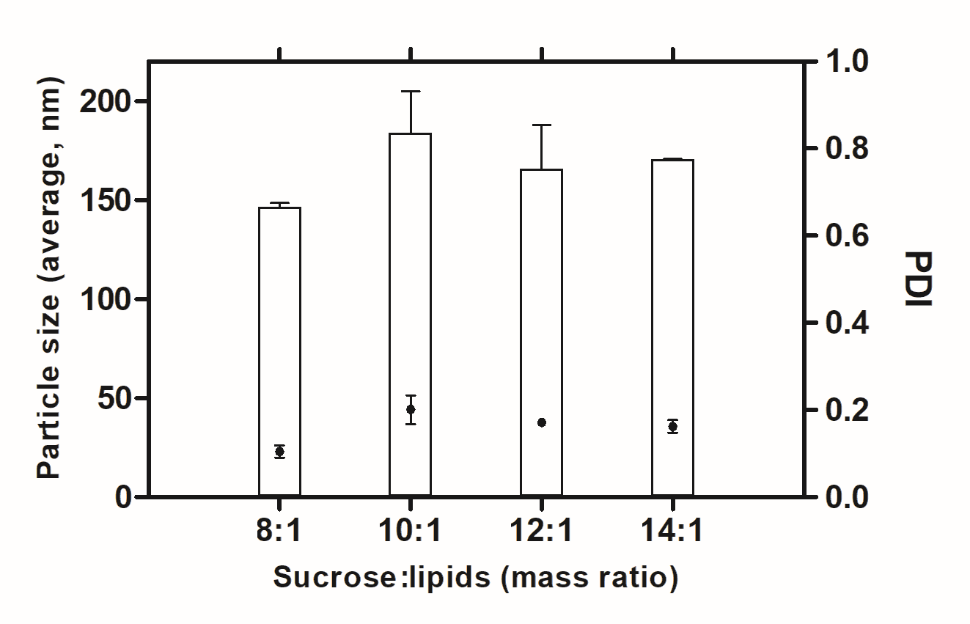
**

**Figure S1.** Influence of high concentration of sucrose in freeze-drying of liposomes. Size (average, nm) and PDI of liposomes in the presence of different sucrose concentrations, after freeze-drying. Values represent the mean + SD of 2 independent experiments.


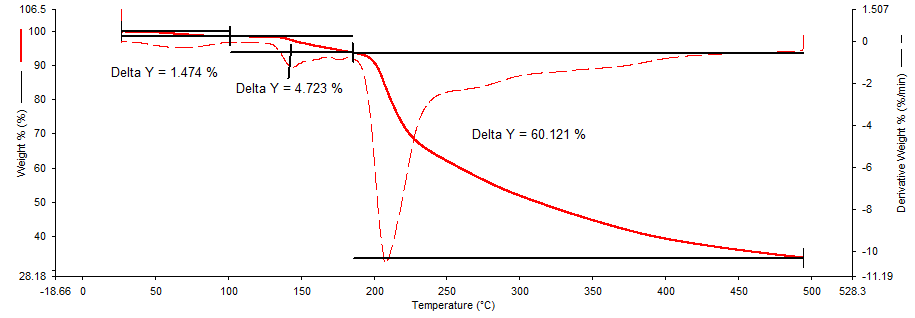


**Figure S2.** Thermogravimetric analysis (TGA) of liposomes containing sucrose (8:1) after the freeze-drying process.

**Table S1.** Influence of sucrose distribution and freezing rate on liposomes encapsulating methotrexate.

| Freezing rate | Distribution | Z-average (nm) | PDI | Leakage |
| --- | --- | --- | --- | --- |
| Quick  (non fixed) | One side | 166.5 ± 4.1 | 0.165 ± 0.053 | 61.1% |
|  | Two size | 591.8 ± 13.8 | 0.272 ± 0.044 | # |
| Slow  (fixed: ~ -1ºC/min) | One size | 181.8 ± 2.8 | 0.209 ± 0.015 | 69.0% |
|  | Two size | 433.0 ± 25.3 | 0.303 ± 0.158 | # |

*# Leakage not determined due to high values of Z-average and PDI.*

*Values represent the mean of ± SD of 2 independent experiments.*
